# Supplementary material for: Resolving the full spectrum of human genome variation using Linked-Reads
Source: Genome Res. 2019 Apr;29(4):635–45. doi: 10.1101/gr.234443.118 (PMC6442396; doi:10.1101/gr.234443.118)
Supplement: Supplemental Material [file supp_gr.234443.118_Supplemental_longranger-2.2.zip › longranger-2.2.tar.bz2!/lr2.2-public/lib/stan/cmdstan/stan/doc/api/html/classstan_1_1mcmc_1_1diag__e__metric.html]

Stan: stan::mcmc::diag\_e\_metric< M, BaseRNG > Class Template Reference


|  |  |
| --- | --- |
|  | Stan  2.5.0  probability, sampling & optimization |


- Main Page
- Related Pages
- Namespaces
- Classes
- Files

- Class List
- Class Index
- Class Hierarchy
- Class Members

All Classes Namespaces Files Functions Variables Typedefs Enumerations Enumerator Friends Macros Pages

- stan
- mcmc
- diag\_e\_metric

Public Member Functions |
List of all members

stan::mcmc::diag\_e\_metric< M, BaseRNG > Class Template Reference

`#include <diag_e_metric.hpp>`

Inheritance diagram for stan::mcmc::diag\_e\_metric< M, BaseRNG >:

|  |  |
| --- | --- |
| Public Member Functions | |
|  | diag\_e\_metric (M &m, std::ostream \*e) |
|  | |
|  | ~diag\_e\_metric () |
|  | |
| double | T (diag\_e\_point &z) |
|  | |
| double | tau (diag\_e\_point &z) |
|  | |
| double | phi (diag\_e\_point &z) |
|  | |
| const Eigen::VectorXd | dtau\_dq (diag\_e\_point &z) |
|  | |
| const Eigen::VectorXd | dtau\_dp (diag\_e\_point &z) |
|  | |
| const Eigen::VectorXd | dphi\_dq (diag\_e\_point &z) |
|  | |
| void | sample\_p (diag\_e\_point &z, BaseRNG &rng) |
|  | |
| Public Member Functions inherited from stan::mcmc::base\_hamiltonian< M, diag\_e\_point, BaseRNG > | |
|  | base\_hamiltonian (M &m, std::ostream \*e) |
|  | |
|  | ~base\_hamiltonian () |
|  | |
| double | V (diag\_e\_point &z) |
|  | |
| double | H (diag\_e\_point &z) |
|  | |
| virtual void | init (diag\_e\_point &z) |
|  | |
| virtual void | update (diag\_e\_point &z) |
|  | |

|  |  |
| --- | --- |
| Additional Inherited Members | |
| Protected Member Functions inherited from stan::mcmc::base\_hamiltonian< M, diag\_e\_point, BaseRNG > | |
| void | write\_error\_msg\_ (std::ostream \*error\_msgs, const std::exception &e) |
|  | |
| Protected Attributes inherited from stan::mcmc::base\_hamiltonian< M, diag\_e\_point, BaseRNG > | |
| M & | model\_ |
|  | |
| std::ostream \* | err\_stream\_ |
|  | |

## Detailed Description

### template<typename M, typename BaseRNG> class stan::mcmc::diag\_e\_metric< M, BaseRNG >

Definition at line 16 of file diag\_e\_metric.hpp.

## Constructor & Destructor Documentation

template<typename M , typename BaseRNG >

|  |  |  |  |
| --- | --- | --- | --- |
| stan::mcmc::diag\_e\_metric< M, BaseRNG >::diag\_e\_metric | ( | M & | *m*, |
|  |  | std::ostream \* | *e* |
|  | ) |  |  |

inline

Definition at line 20 of file diag\_e\_metric.hpp.

template<typename M , typename BaseRNG >

|  |  |  |  |  |
| --- | --- | --- | --- | --- |
| stan::mcmc::diag\_e\_metric< M, BaseRNG >::~diag\_e\_metric | ( |  | ) |  |

inline

Definition at line 22 of file diag\_e\_metric.hpp.

## Member Function Documentation

template<typename M , typename BaseRNG >

|  |  |  |  |  |  |
| --- | --- | --- | --- | --- | --- |
| const Eigen::VectorXd stan::mcmc::diag\_e\_metric< M, BaseRNG >::dphi\_dq | ( | diag\_e\_point & | *z* | ) |  |

inlinevirtual

Implements stan::mcmc::base\_hamiltonian< M, diag\_e\_point, BaseRNG >.

Definition at line 39 of file diag\_e\_metric.hpp.

template<typename M , typename BaseRNG >

|  |  |  |  |  |  |
| --- | --- | --- | --- | --- | --- |
| const Eigen::VectorXd stan::mcmc::diag\_e\_metric< M, BaseRNG >::dtau\_dp | ( | diag\_e\_point & | *z* | ) |  |

inlinevirtual

Implements stan::mcmc::base\_hamiltonian< M, diag\_e\_point, BaseRNG >.

Definition at line 35 of file diag\_e\_metric.hpp.

template<typename M , typename BaseRNG >

|  |  |  |  |  |  |
| --- | --- | --- | --- | --- | --- |
| const Eigen::VectorXd stan::mcmc::diag\_e\_metric< M, BaseRNG >::dtau\_dq | ( | diag\_e\_point & | *z* | ) |  |

inlinevirtual

Implements stan::mcmc::base\_hamiltonian< M, diag\_e\_point, BaseRNG >.

Definition at line 31 of file diag\_e\_metric.hpp.

template<typename M , typename BaseRNG >

|  |  |  |  |  |  |
| --- | --- | --- | --- | --- | --- |
| double stan::mcmc::diag\_e\_metric< M, BaseRNG >::phi | ( | diag\_e\_point & | *z* | ) |  |

inlinevirtual

Implements stan::mcmc::base\_hamiltonian< M, diag\_e\_point, BaseRNG >.

Definition at line 29 of file diag\_e\_metric.hpp.

template<typename M , typename BaseRNG >

|  |  |  |  |
| --- | --- | --- | --- |
| void stan::mcmc::diag\_e\_metric< M, BaseRNG >::sample\_p | ( | diag\_e\_point & | *z*, |
|  |  | BaseRNG & | *rng* |
|  | ) |  |  |

inlinevirtual

Implements stan::mcmc::base\_hamiltonian< M, diag\_e\_point, BaseRNG >.

Definition at line 43 of file diag\_e\_metric.hpp.

template<typename M , typename BaseRNG >

|  |  |  |  |  |  |
| --- | --- | --- | --- | --- | --- |
| double stan::mcmc::diag\_e\_metric< M, BaseRNG >::T | ( | diag\_e\_point & | *z* | ) |  |

inlinevirtual

Implements stan::mcmc::base\_hamiltonian< M, diag\_e\_point, BaseRNG >.

Definition at line 24 of file diag\_e\_metric.hpp.

template<typename M , typename BaseRNG >

|  |  |  |  |  |  |
| --- | --- | --- | --- | --- | --- |
| double stan::mcmc::diag\_e\_metric< M, BaseRNG >::tau | ( | diag\_e\_point & | *z* | ) |  |

inlinevirtual

Implements stan::mcmc::base\_hamiltonian< M, diag\_e\_point, BaseRNG >.

Definition at line 28 of file diag\_e\_metric.hpp.

---

The documentation for this class was generated from the following file:

- src/stan/mcmc/hmc/hamiltonians/diag\_e\_metric.hpp


---

[ Stan Home Page ]

*© 2011–2014,
Stan Development Team.*
